# Rapid eye movement sleep behavior disorder and its relation to Parkinson's disease: The potential of graph measures as brain biomarkers to identify the underlying physiopathology of the disorder

**DOI:** 10.1002/brb3.3460

**Published:** 2024-03-17

**Authors:** Milad Najafzadeh, Fatemeh Mohammadian, Sara Mirabian, Zohre Ganji, Hossein Akbari, Masoud Rezaie, Esmaeil Ranjbar, Hoda Zare, Shahrokh Nasseri, Luigi Ferini‐Strambi

**Affiliations:** ^1^ Department of Medical Physics, Faculty of Medicine Mashhad University of Medical Sciences Mashhad Iran; ^2^ Department of Anatomy and Cell Biology, School of Medicine Mashhad University of Medical Sciences Mashhad Iran; ^3^ Medical Physics Research Center Mashhad University of Medical Sciences Mashhad Iran; ^4^ Vita‐Salute San Raffaele University Milan Italy; ^5^ Division of Neuroscience, Sleep Disorders Center San Raffaele Scientific Institute Milan Italy

**Keywords:** Brain, Imaging, Parkinson's disease, sleep

## Abstract

Rapid eye movement behavior disorder (RBD) is a parasomnia characterized by the loss of skeletal muscle atonia during the rapid eye movement (REM) sleep phase. On the other hand, idiopathic RDB (iRBD) is considered the prelude of the various α‐synucleinopathies, including Parkinson's disease (PD), dementia with Lewy bodies and multiple system atrophy. Consequently, over 40% of patients eventually develop PD. Recent neuroimaging studies utilizing structural magnetic resonance imaging (s‐MRI), diffusion‐weighted imaging (DWI), and functional magnetic resonance imaging (fMRI) with graph theoretical analysis have demonstrated that patients with iRBD and Parkinson's disease have extensive brain abnormalities. Thus, it is crucial to identify new biomarkers that aid in determining the underlying physiopathology of iRBD group. This review was conducted systematically on the included full‐text articles of s‐MRI, DWI, and fMRI studies using graph theoretical analysis on patients with iRBD, per the procedures recommended by Preferred Reporting Items for Systematic Reviews and Meta‐Analyses (PRISMA). The literature search was conducted through the PubMed and Google scholar databases concentrating on studies from September to January 2022. Based on the three perspectives of integration, segregation, and centrality, the reviewed articles demonstrated that iRBD is associated with segregation disorders in frontal and limbic brain regions. Moreover, this study highlighted the need for additional longitudinal and multicenter studies to better understand the potential of graph metrics as brain biomarkers for identifying the underlying physiopathology of iRBD group.

## 1 INTRODUCTION

Rapid eye movement sleep behavior disorder (RBD) is a parasomnia characterized by the loss of skeletal muscle atonia during the sleep REM phase resulted to dream‐enacted behaviors including thrashing, punching, and kicking (Gnarra et al., 2023). A meta‐analysis reported that the prevalence of this disorder in a normal population ranges from 0.68% to 5.65 %; however, a plethora of cases still go unrecognized (Cicero et al., 2021). RBD pathogenicity is thought to be associated with dysfunction of the lower nuclei of the brainstem that regulate muscle atonia during sleep (Campabadal et al., 2021).

When the sleep disorder is isolated with no clinical symptoms of a nervous disorder, it is referred to as idiopathic RBD (iRBD). The importance of iRBD is on the rise as it is considered a prodromal α‐synuclein syndrome caused by neurodegeneration (Kalinderi et al., 2024). A considerable majority of iRBD patients (about 73.5%) ultimately develop Parkinson's disease (PD), Lewy bodies (DLB), and multiple system atrophy during a 12‐year follow‐up (Gnarra et al., 2023). Thus, it is logical to expect malfunctions in structures other than the nuclei of the brainstem, including the striatum, substantia nigra, the limbic system, and the cerebral cortex during the development of this disorder (Campabadal et al., 2021). Within the PD population, estimates show the presence of RBD symptoms in addition to classical motor complications, and the prevalence of RBD in PD ranges from 35% to 50% (Baumann‐Vogel et al., 2020). Evidence also indicates that RBD may be observed in the PD prodromal phase (Schaeffer et al., 2020). Therefore, there is a need to identify brain biomarkers in iRBD and in the PD population with RBD to both better understand the pathogenesis and better diagnose disease progression (Schaeffer et al., 2020).

On the other hand, there is currently no effective treatment to delay the neurodegenerative process in people suffering from Parkinson's disease, probably because the neurodegenerative process is too advanced at the time of diagnosis (Rizek et al., 2016). Patients are typically treated symptomatically, with an emphasis on relieving motor symptoms (such as tremors, stiffness, and bradykinesia) as well as nonmotor symptoms (such as constipation, cognition, mood, and sleep) (Rizek et al., 2016). Thus, in order to test neuroprotective agents to postpone or prevent the beginning of PD, early diagnosis of iRBD patients is crucial (de Natale et al., 2022).

A biomarker is a measurable characteristic indicating normal or pathogenic biological processes or pharmacological responses to therapeutic drugs (Miglis et al., 2021). Biomarkers used for diagnosis of iRBD patients include neurophysiological (polysomnography, actigraphy, electroencephalogram), clinical (hyposmia, cognitive dysfunction, visual dysfunction, motor function, autonomic function) and biological biomarker (blood test, CSF, tissue biopsy), neuroimaging (positron emission tomography [PET], single‐photon emission computed tomography [SPECT], structural magnetic resonance imaging [MRI], DWI and fMRI), and genetic biomarker (GBA variants, SNCA, TMEM175) (Bramich et al., 2022; Gnarra et al., 2023; Zhang et al., 2023).

Polysomnography is the only diagnostic technology that can be utilized for definite diagnosis of iRBD, but it is not commonly available. Furthermore, screening a large number of people is not feasible (de Natale et al., 2022; Gnarra et al., 2023). Less complex instruments like the RBD screening questionnaire (RBDSQ) have been developed. While the RBDSQ is sensitive enough to detect diseases, it might mistakenly identify people who have disorders similar to iRBD, particularly those who have severe sleep apnea, sleep terrors, or sleepwalking (de Natale et al., 2022).

Actigraphy is a noninvasive method that can be used for assessment of sleep disorders (Bramich et al., 2022; Liguori et al., 2023). Measured biomarkers such as increased daytime nap frequency, sleep‐wake cycle dysregulation, and diurnal motor hypoactivity can be used for early diagnosing of patients with iRBD from healthy control, PD, restless leg syndrome (RLS) and sleep apnea (Liguori et al., 2023). However, due to the limited number of investigations in patients with iRBD, the potential use of this method as predictor of disease progression in iRBD needs to be further investigated (Liguori et al., 2023).

Analysis of EEG data have revealed that those with iRBD who were in the risk of developing PD over a 3‐ to 4‐year follow‐up had greater slow to fast power ratios, disruptions in *θ* waves, and increased *δ* and *θ* waves in the cerebral cortex in comparison to the healthy control (Bergmann et al., 2024; de Natale et al., 2022). Additionally, slowing of signals in the temporal and occipital lobes has been noted (de Natale et al., 2022). In the next future, EEG data can be a valid diagnostic and prognostic biomarker of α‐synucleinopaty and will gain more attentions using artificial intelligence and machine learning‐based methods (Miglis et al., 2021).

Previous studies showed that cognitive impairment can serve a potential biomarker of phenoconversionin iRBD and inclusion of cognitive assessment in standard evaluations of iRBD seems to be beneficial (de Natale et al., 2022; Wang et al., 2022). However further studies are needed to choose the type of test as well as to identify the criteria for defining mild cognitive impairment (MCI) (Fiamingo et al., 2023).

Clinical biomarkers including hyposmia, cognitive dysfunction and visual dysfunction are associated with increased risk of developing PD in patients with iRBD but having low specificity has limited their clinical use as early biomarker of diagnosing iRBD (de Natale et al., 2022).

Given that emerging of motor abnormalities occurs at the latest stages of prodromal phase in patients with iRBD, quantitative assessment of motor function can be a powerful prognostic and monitoring biomarker of α‐synucleinopathy (Miglis et al., 2021; Wang et al., 2022). Promising findings have been observed regarding the use of novel technologies for investigating motor functions in patients with iRBD such as leg movement during REM, postural and rest tremor, voice and speech features, and pupillary responses (Barber et al., 2023; Gnarra et al., 2023). However, their use as a potential biomarker of PD risk needs to be further tested (Gnarra et al., 2023). Autonomic dysfunction, a common nonmotor symptoms in iRBD patients, includes decreased heart rate variability, urinary dysfunction, sexual and erectile dysfunction, orthostatic hypotension. Autonomic abnormalities can serve a potential diagnostic markers of α‐synucleinopathy but the role as biomarker of disease's progression needs to be further tested (Figoril, 2023; Šonka, 2022).

Findings have shown that individuals with iRBD have a higher frequency of mutations in the genes GBA and TMEM175 as well as lower expression of the SNCA gene (Arnaldo et al., 2023; de Natale et al., 2022). Nonetheless, additional analysis of these findings in a cohort study is required.

The effect of neuroimaging on the timely diagnosis of iRBD has been well known (Grimaldi et al., 2023; Valli et al., 2022). Two most important neuroimaging methods are molecular imaging and MRI (Campabadal et al., 2021; Valli et al., 2022). Biomarkers found in molecular studies include dopaminergic (measured using [^123^I]FP‐CIT, 11C‐donepezil) and cholinergic system (measured using [^11^C]donepezil PET tracer, [^11^C]PK11195 tracer, and [^18^F] FEOBV PET radioligand), blood perfusion, and glucose metabolism (measured using [^99^mTc]ECD and [^123^I]IMP SPECT tracers]) (Arnaldi et al., 2021; Valli et al., 2022). Molecular imaging specially ^123^I‐FP‐CIT SPECT can be a potential biomarker of disease progression in patient with iRBD especially when combined with the clinical features (Arnaldi et al., 2021). However, there are several limitations regarding the use of these methods as biomarker of disease's progression. First, investigated studies for molecular studies have relatively small samples specially in longitudinal studies, thus the results must be interpreted with caution. Second, further longitudinal studies are needed to identify different pattern of radionuclide imaging alterations to distinguish iRBD patients developing to PD. Third, resolution of current PET scanners limits the evaluation of the small brainstem structures. Fourth, using ionizing radiation may limit their use (Miglis et al., 2021; Valli et al., 2022). Thus, MRI can be an attractive alternative. Several biomarkers have found in structural MRI including frontal cortical and basal ganglia atrophy, reduction of neuromelanin in the SN (measured using neuromelanin sensitive imaging), increased iron content in nigrosome1 and cortical thinning in the temporal, orbitofrontal, insular and occipital cortices (Campabadal et al., 2021; Grimaldi et al., 2023).

However, further longitudinal studies in large cohorts are needed to test the reliability of the results (Grimaldi et al., 2023). Regarding the fMRI and DWI, results have a considerable heterogeneity and so more longitudinal investigations need to be performed (Campabadal et al., 2021; Grimaldi et al., 2023). Concerning the investigation of misfolding and aggregation of α‐synuclein, promising findings have been observed but further longitudinal studies are needed (Figoril, 2023; Wang et al., 2022; Zitser et al., 2020).

Individuals with iRBD and PD exhibit a similar longitudinal connectome development pattern, which strengthens the case for employing them as biomarkers for early diagnosis of PD (Campabadal et al., 2021). Disturbance in structural and functional connectivity of the brain in PD patients has been reported in several studies (Bergamino et al., 2020; Sanjari Moghaddam et al., 2020). Moreover, some progressive disorders in PD may reflect changes in the integration and segregation of distributed brain networks (Prajapati & Emerson, 2021); consequently, information integration and segregation capacities decline between different brain regions.

The graph theory provides tools to briefly quantify the characteristics of complex networks and characterizes the interactions (represented by edges) between different brain regions (represented by nodes) (Bullmore & Sporns, 2009; Semmel et al., 2022). This analysis investigates all structures of the network by providing a simple model of brain connectivity represented by nodes and edges (Bullmore & Sporns, 2009; Semmel et al., 2022). The graph theory analysis allows the study of brain areas and their different networks by decreasing the complex structure of the brain network to a set of parameters that characterize the network topological measure (Bullmore & Sporns, 2009; Semmel et al., 2022). Furthermore, promising findings suggest that the extraction of topological measures from any imaging modality using graph theory analysis may improve clinical interpretability (Nigro et al., 2022; Semmel et al., 2022). As yet, the graph theory was mainly used to describe brain diagrams obtained with anatomical, morphological, and functional connectivity neuroimaging techniques because it is difficult to obtain a detailed description of human brain connectivity. It is argued that graphs made from different imaging modalities should have the related network topologies if they reflect real brain connectivity.

Structural imaging modalities and functional connectivity in iRBD have been thoroughly reviewed recently, but no relationship was established between the features extracted from this theory using various imaging modalities and clinical findings (Campabadal et al., 2021; Valli et al., 2022). Given the clinical importance of iRBD and the possible observation of this disorder in the PD initial phase, the discovery of common brain biomarkers can significantly contribute to the detection and selection of appropriate therapeutic strategies. Therefore, this review study aimed to evaluate graph features at two local and global levels and to assess the potential of these metrics as brain biomarkers to identify the underlying physiopathology of iRBD group.

## 2 METHODS

### 2.1 Study selection

This systematic review article is based on the PRISMA guideline. The starting point of this systematic review was a protocol in which research questions and strategies were determined to reduce research expectations. To achieve the study objective (comparison of both groups), papers related to structural MRI and functional connectivity in iRBD was searched in the PubMed database and Google Scholar in September 2022. The following word combinations were used for the search: “graph theory,” “graph analysis,” “network analysis,” “connectome,” “connectomics,” iRBD, structural MRI(s‐MRI), diffusion‐weighted imaging (DWI), diffusion tensor imaging (DTI), functional MRI (fMRI), structural connectivity, and functional connectivity.

The following criteria were then applied for the inclusion in this review: (1) main articles, (2) English full text is available, (3) case‐control studies consisting of iRBD patient and graph analysis, and (4) structural and functional connectivity MRI studies using neuroimaging analysis software.

The following information was extracted from each study: (1) author(s), (2) publication year, (3) source of data, (4) sample size, (5) population data, (6) graph features, (7) imaging modality, and (8) brain area.

**FIGURE 1:**
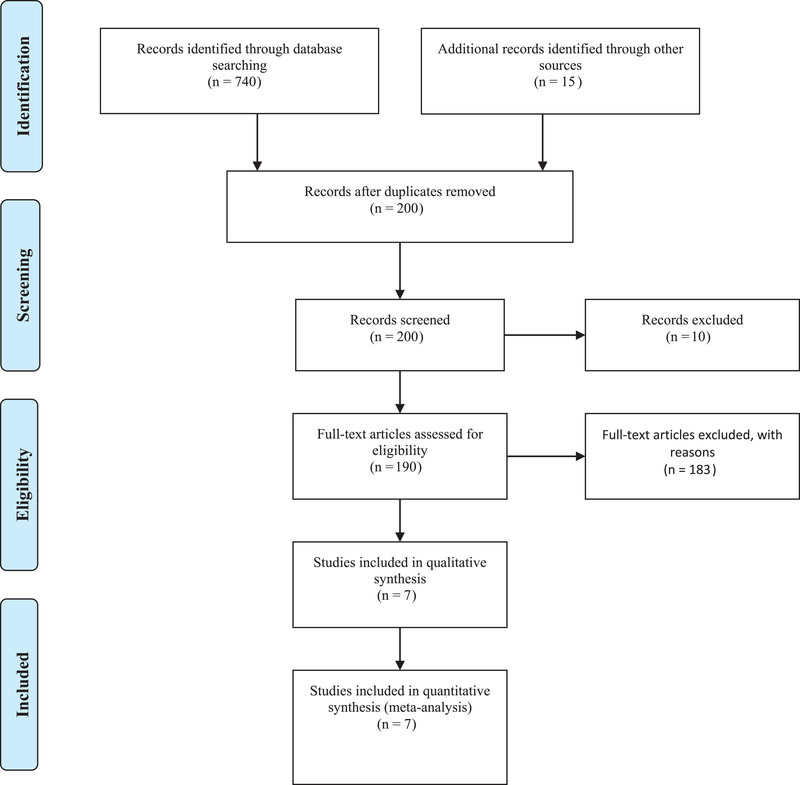
PRISMA 2009 flow diagram.

Figure 1 depicts the PRISMA diagram. Studies published in English were reviewed using the presented keywords and inclusion criteria in iRBD patient compared to healthy controls. Exclusion criteria were the following: (a) studies other than neuroimaging including positron emission tomography and electroencephalography studies, (b) animal studies, (c) conference proceedings or abstracts, and (d) articles in other languages.

### 2.2 Data extraction

The following variables were recorded from extracted articles: (a) population characteristics (sample size, age, gender, and year), (b) graph features, (c) brain areas, (d) imaging parameters, (e) imaging processing parameters to produce the graph network, and (f) associations between clinical data and graph parameters.

### 2.3 Graph theoretical concepts

In graph theory, a network is defined as a set of nodes and edges or their linear relationship (Barber et al., 2023). In all brain graphs, nodes represent anatomical areas of the body. In studies on an MRI‐derived network, a common approach for node representation is to parcellate coregistered anatomical MRI using a validated scheme. These schemes determine the network size and are specified based on anatomical landmarks, for example, AAL (Rolls et al., 2020).

Edges represent structural or functional connections between different brain areas (Bullmore & Sporns, 2009). In s‐MRI, the edge is determined by the connection between brain areas using covariance models, including the morphological connections of structural anatomical features (Wu et al., 2021). Connectivity between nodes is obtained using the tracts of brain white matter in DWI, which is sensitive to the diffusion of water molecules (Wu et al., 2021). In fMRI imaging, which measures the blood oxygen dependent level (BOLD) signal level, the time connection between brain nodes determines edges (Wu et al., 2021).

Ultimately, graphs can be divided into three groups: weighted, threshold weighted, and nonweighted (binary). Unlike binary graphs, weighted networks contain information about the connectivity strength between brain areas, which include weak connections and may cause noise entry into the network (Adebisi & Veluvolu, 2023). Table 1 summarizes the graph important features measured in iRBD studies.

**TABLE 1:** Graph terminology.

Parameters	Definition
Hub	A node with high centrality
Small‐worldness	It describes the ratio between clustering coefficient and mean of characteristics path length. It balances integration and segregation in a network while decreasing energy consuming.
Measures of integration	
Global efficiency (GE)	It measures parallel information transfer in a network.
Characteristic path length (CPL)	The approximate number of connections (edges) that must be passed to connect two two nodes. It indicates the ease of sharing information between regions in the network.
Assortativity coefficient (AC)	It expresses the tendency of nodes to connect with other areas with the same number of connections (edges).
Measures of segregation	
Clustering coefficient (CC)	Percentage of neighbors of a given node that are neighbors to each other. It shows the tendency of nodes to communicate with the adjacent nodes.
Local efficiency (LE)	It is a measure of the average efficiency of information transfer within local subgraphs or local neighbors, and it is defined as the shortest average path length of all neighbors of a certain anatomical region among themselves (same as GE).
Nodal shortest path length (SPL)	The shortest path length between two nodes in a graph is the path with the least number of connections (edges).
Measures of centrality	
Betweeness centrality (BC)	For a given anatomical region, it determines its effect on the flow of information between other regions.
Nodal degree centrality (DC)	The centrality of a node expresses the number of its connections (edges).

### 2.4 Network integration, segregation, centrality, and small‐worldness

Graph theory parameters express the balance between network integration and network segregation (Bullmore & Sporns, 2009). Integration denotes the process level of investigated information in the brain in which data processes in scattered spatial regions. In this article, the most common integration metrics were global efficiency (GE), characteristic path length (CPL), and assortativity coefficient (AC). GE measures parallel information transfer in a network. CPL is the approximate number of edges that should be passed to connect two brain areas (nodes), indicating the ease of sharing information between areas in the network. The relationship between the two mentioned parameters is defined as GE to be the harmonic inverse of CPL. Higher levels of GE and CPL indicate greater integration of brain networks. Assortativity represents the tendency of nodes to establish connectivity with other areas with a similar number of edges (Rubinov & Sporns, 2010)

Segregation represents the level of the process of investigated information locally in self‐contained regions of the brain (Bullmore & Sporns, 2009). In this article, the most common graph metrics were clustering coefficient (CC), Local Efficiency (LE), and nodal SPL, which quantify segregation features. CC delineates information processing in the network in local brain regions. Similar to GE, LE reflects information transfer efficiency at the local level (self‐contained regions of the brain) in the network (Bullmore & Sporns, 2009). A path with the minimum number of edges is the shortest path length between two brain regions in a graph. Higher values for these parameters indicate more segregation in the network (Bullmore & Sporns, 2009).

Centrality parameters determine the importance of an anatomic region (node) inside a network based on its connections to adjacent regions. The most important centrality parameters include degree centrality (DC: the number of edges connected to an anatomic region) and betweenness centrality (BC: a percentage of the shortest path in the network that encompasses that region) (Rubinov & Sporns, 2010).

Based on the segregation and centrality networks, it is also possible to calculate another feature known as small‐worldness (Rubinov & Sporns, 2010). This feature is known for high local processing with lower remote projection. In the graph analysis, small‐worldness is defined as the ratio of the clustering coefficient to the average characteristic path length compared to a random network (Bullmore & Sporns, 2009).

## 3 RESULTS

The search in the database retrieved 740 articles, about 200 of which remained after duplicate elimination. Excluded articles (*n* = 100) concerned animal studies (*n* = 10), studies other than neuroimaging (*n* = 60), articles in other languages (*n* = 10), and review articles (*n* = 20). Next, articles were evaluated for inclusion eligibility, and 93 articles were excluded due to the lack of studies on structural and functional connectivity between patient and HC groups. Finally, the included studies were 7 articles, namely, 138 and 126 individuals in HC and iRBD groups, respectively. Data extracted from articles in this review study are presented in Tables 2, 3, 4.

**TABLE 2:** Studies of structural and functional MRI in iRBD.

Study	Sample	Demographics	Modality	Graph features	Contrast	Result	Brain region
Park et al. (2019)	10 iRBD: 6 M, 4 F 14 HC, 8 M, 6 F	67.0 ± 6.4 67.2 ± 4.5	s‐MRI	Average degree, CPL mean CC GE LE Small‐worldness index BC Hubs	iRBD < HC iRBD > HC	AD* GE* Global LE* Mean CC Small‐worldness CPL* BC*	iRBD vs. HC ↑ BC L caudate nucleus, M frontal gyrus, Lateral orbitofrontal and superior frontal cortex R caudate Pars triangularis, Rostral M frontal ↓ BC Pars orbitalis in the R hemisphere Recruited hubs Local brain network including caudate nucleus Frontal cortex
Lee et al. (2022)	20 iRBD, 11 M, 9F 20 HC, 7 M, 13F	68.8 ± 6.7 65.9 ± 6.9	DWI	Mean CC GE LE CPL Small‐worldness index AC BC	iRBD = HC iRBD > HC	Mean CC GE Global LE CPL Small‐worldness index AC BC*	iRBD vs. HC: ↑ BC L olfactory cortex in the temporal lobe L precentral gyrus in the frontal lobe L supramarginal gyrus: parietal lobe L temporal pole, superior temporal gyrus Lobule of X of vermis L crus II of cerebellar hemisphere
Wen et al. (2017)	20 iRBD, 6 M, 14 F 106 PD, 67 M, 39 F 55 HC. 38 M, 17F	61.24 ± 9.85 68.26 ± 5.71, 6.14 ± 6.56 61.03 ± 10.71	DWI	Network density CC CPL, Small‐worldness, GE AC	iRBD > HC,PD iRBD > PD iRBD < HC iRBD = HC = PD	Small‐worldness * GE CC AC AC CPL	iRBD vs. HC, PD ↑ small‐worldness, CC: WM structural network Local connectivity between multiple regions relating to motor, sleep, and olfactory functions iRBD vs. HC ↑ connectivity between the parahippocampus: medial temporal lobe> Cerebellum Between the SMA area and putamen
Campabadal et al. (2020)	20 iRBD, 14 M, 6F 27 HC, 13 M, 14F	71 ± 10 66.5 ± 13	Rs‐fMRI	Global CC Local CC Global node degree local node degree Small‐worldness Path length LE BC	iRBD < HC iRBD = HC	BC Global CC Local CC Global node degree local node degree Small‐worldness SPL Global LE	iRBD vs. HC ↓ BC L superior parietal lobule
Chen et al. (2022)	21 iRBD 23 PD 22 HC	61 ± 10.68 60.13 ± 11.22 60.27 ± 7.6	Rs‐fMRI	Small‐worldness, GE LE Nodal DC Nodal LE Nodal SPL	iRBD, nRBD‐PD < HC iRBD > HC iRBD < HC	GE Nodal LE* Small‐worldness CPL Nodal SPL *	iRBD vs. HC ↓nodal SPL Precentral gyrus Postcentral gyrus Supramarginal gyrus superior temporal gyrus Supra‐motor area Straight gyrus Middle cingulate gyrus Rolandic operculum. ↓ nodal LE L lingual gyrus L R superior occipital gyrus RMoccipital gyrus, ↓DC R anterior cingulate para‐cingulate gyri L R anterior cingulate R cuneus L and R superior Occipital gyrus L and R M occipital gyrus
Geng et al. (2022)	21 iRBD, 14 M, 7F 22 HC, 9 M,12F	61.00 ± 10.68 60.27 ± 7.60	Rs‐fMRI	CC CPL Normalized CC Normalized CPL Small‐worldness index GE Nodal LE DC Nodal LE Nodal SPL	iRBD < HC iRBD = HC	Nodal LE Nodal SPL Small‐worldness	iRBD vs. HC: ↓ nodal LE L lingual gyrus R M occipital gyrus BL superior occipital gyrus ↓ nodal shortest path length L M cingulum gyrus supramarginal gyrus Paracentral lobule R inferior frontal triangular gyrus Rectus gyrus Rolandic operculum BL postcentral gyrus Precentral gyrus Superior temporal gyrus
Moodie et al. (2018)	15 PD 14 iRBD 13 HC	60.0 ± 11.5 (for all groups)	Rs‐fMRI	Connectivity strength	**RBD, PD < HC**	Connectivity strength *	Not stated

Abbreviations: HC, healthy control; AD, average degree; GE, global efficiency; LE, local efficiency; CC, clustering coefficient; CPL, characteristic path length; BC, betweenness centrality; AC, assortativity coefficient; L, left; R, right; M, middle; BL, bilateral; iRBD, idiopathic rapid eye movement sleep behavior disorder.

*Significant change between two groups.

**TABLE 3:** Data acquisition and graph construction for structural studies in iRBD.

Study	Modality	Source of imaging data	Number of slice	Slice thickness (mm)	Parcellation scheme	Number of diffusion direction and *b*0	Tractography method	Network construction method	software	Weighted or unweighted graph
Park et al. (2019)	s‐MRI (T1, T2‐FLAIR)	Single center (single hospital)	Not stated	Not stated	(73 nodes, 60 cortical and 13 subcortical regions)	–	–	Node: anatomical regions (cortical volume) Edge: partial correlation coefficients between every pair of brain regions	Freesurfer BRAPH	Weighted
Lee et al. (2022)	DWI (Spin‐echo EPI)	Single center (single hospital)	Not stated	2.25 mm	AAL (90 nodes)	32 (*b* = 0 and *b* = 1000)	Not stated	Node: anatomical regions Edge: fiber density	DSI studio	Weighted
Wen et al. (2017)	DWI (2‐dimensional echo‐planar DTI sequence)	PPMI	72	2 mm	AAL(116 cortical and subcortical regions)	64 (*b* = 0 and *b* = 1000)	Deterministic (QSDR)	Node: anatomical regions Edge: product of the count of the connection tract	DSI Studio Brain Connectivity Toolbox	Weighted

**TABLE 4:** Data acquisition and graph construction for functional studies in iRBD.

Study	Modality	Source of imaging data	Number of image volume	Slice thickness (mm)	Smoothing kernel size and band pass filter	Parcellation scheme	Motion correction	Network construction method	Software	Weighted or unweighted graph
Campabadal et al. (2020)	Eye closed Rs‐fmri	Single center (sleep unit)	240	3	Not stated	Brainnetome Atlas (246 nodes)	ICA‐AROMA	Nodes: anatomical regions Edge: not stated	AFNI tools BCT	Not stated
Chen et al. (2022)	Rs‐fmri	Single center (single hospital)	210	4	4 mm 0.01−0.08	AAL (90 nodes)	Not stated	Nodes: anatomical regions Edge: interaction between each two brain regions	DPARSF GRETNA	Weighted
Geng et al. (2022)	Rs‐fmri	Single center (single hospital)	Not stated	4	Not stated	AAL (90 nodes)	Not stated	Nodes: anatomical regions Edge: Pearson's correlation coefficients between the mean time series of all possible pairs	DPARSF GRETNA	Not stated

### 3.1 Brain network disruption in iRBD

Table 2 shows the results derived from the seven extracted studies in which graph features between control (HC), iRBD, group is reported using s‐MRI, DTI, and rs‐fMRI data. Table 3 represents imaging data used for making the graph network, and software used in the reviewed studies. As shown in Table 2, integration and segregation are the extracted features in most studies. Hub distribution was reported in four studies. In this section, the findings from the reviewed studies are described in more detail.

### 3.2 Graph and connectivity changes in iRBD

The results of this study for both patient and HC groups are shown in Table 2. Reductions in the global features of structural connectivity compared to HC were reported in two studies in the iRBD group (Park et al., 2019; Wen et al., 2017). However, increases in the global features of structural connectivity were observed in the iRBD group in two studies (Park et al., 2019; Wen et al., 2017), (Guo et al., 2018). For structural connectivity, elevated local features in the iRBD group were claimed in three studies (Lee et al., 2022; Park et al., 2019; Wen et al., 2017). Reduced local features (Park et al., 2019) of structural connectivity in the iRBD group were announced in one investigation.

The local features of functional connectivity decreased reportedly in the iRBD group in three studies. One research reported an increase in the global features of functional connectivity in the iRBD group (Table 2). Moreover, rs‐fMRI was used in one study, which reported a reduction in connectivity in iRBD group compared to the HC group (Moodie et al., 2018) (Table 2).

Most of the involved areas are located in the frontal and parietal lobes; there is also impaired connectivity between lobes in these areas. Additionally, reduced connectivity in the iRBD group compared to the HC was denoted in one investigation, which may be a direct result of impaired connectivity in the cortico‐cortical region.

### 3.3 CC in iRBD

In seven studies extracted in this review (Table 2), CC was obtained for different brain areas in iRBD group compared to HC in eight investigations. Among these, four studies were related to iRBD group (Campabadal et al., 2020; Lee et al., 2022; Park et al., 2019; Wen et al., 2017)

According to the main findings (Table 2), a reduction and an increase in mean CC were reported for the iRBD group compared to HC in one (s‐MRI) (Wen et al., 2017) and another study (DWI), respectively. Wen et al. (2017) also found a significant difference between the two mentioned groups. In the iRBD group, small‐worldness was also denoted in six studies (Campabadal et al., 2020; Geng et al., 2022; Lee et al., 2022; Park et al., 2019; Wen et al., 2017). In two studies (Campabadal et al., 2020; Wen et al., 2017), this feature was calculated compared to a random network, and no differences were observed between iRBD and HC groups (Table 2). Park et al. (2019) and Chen et al. (2022) obtained a reduction in this index compared to HC (lower segregation), but Wen et al. (2017) found an increase (higher segregation) in this index in comparison with HC.

### 3.4 CPL in iRBD

The results obtained from the reviewed studies in this systematic review (Table 2) indicate that the results reported for CPL are not stable and identical in all studies. In the iRBD group, an increase in CPL using s‐MRI and fMRI imaging techniques in two studies (Chen et al., 2022; Park et al., 2019), suggesting that it moves away from a random network, with elevated integration. Park et al. (2019) detected a significant difference between the two groups using s‐MRI imaging.

### 3.5 GE in iRBD

GE was denoted in the iRBD group compared to HC in six studies. In comparison with HC, a decrease in GE was reported in two studies (Chen et al., 2022; Park et al., 2019) (one using s‐MRI and one using rs‐fMRI) indicating the lower integration, among which one study (Park et al., 2019) found a significant reduction. Furthermore, Lee et al. (2022) reported no differences between iRBD and HC groups using DWI. In contrast, Wen et al. (2017) used DWI and found that there was higher GE in iRBD patients compared to HC. The GE was also stated in one study without any calculation (Geng et al., 2022) (Table 2).

### 3.6 LE in iRBD

Five studies evaluated LE in the iRBD group compared to HC, among which LE was estimated globally and locally in three (Campabadal et al., 2020; Lee et al., 2022; Park et al., 2019) and two (Chen et al., 2022; Geng et al., 2022) investigations, respectively. At the global level, HC and iRBD groups were not different in two studies (Campabadal et al., 2020; Lee et al., 2022), one using DWI and another using rs‐fMRI, but Park et al. (2019) recognized a decrease in this parameter in comparison between the two groups, showing reduced segregation (see Table 2). In addition, the two studies (Chen et al.,^2022^; Geng et al., 2022) reported a direct correlation between the TMT‐A score and LE middle occipital gyrus in the iRBD group. Using rs‐fMRI, Rubinov and Sporns (2010) identified a significant decline in LE in brain regions located at occipital lobe including middle occipital gyrus, superior occipital gyrus, calcarine fissure and left lingual gyrus. Likewise, Park et al. (2019) noticed significantly reduced LE in brain occipital regions (left lingual gyrus and right middle occipital) using rs‐fMRI. (Chen et al., 2022).

### 3.7 AC in iRBD

In the iRBD group, AC was estimated in two surveys (Lee et al., 2022; Wen et al., 2017). Lee et al. (2022) calculated AC using DWI and found a greater AC in iRBD group patients than in HC. In contrast, Wen et al. (2017) reported a decrease in this index. (Li et al., 2020). The results revealed that integration rose in the iRBD group.

### 3.8 BC in iRBD

BC was assessed in the iRBD group (Campabadal et al., 2020; Lee et al., 2022; Park et al., 2019), including the extraction of this parameter from s‐MRI, DWI, and rs‐fMRI. Using s‐MRI, increasing BC in the left caudate nucleus of the brain was reported by Park et al. (2019) in the caudate nucleus and frontal lobe. After this research, Lee et al. (2022) found an increase in the left olfactory cortex, left precentral gyrus, superior temporal gyrus and vermis (see Table 2). Both studies noticed a significant difference in the BC parameter between the two groups. Conversely, this parameter reportedly decreased in the left superior parietal lobule of the brain using rs‐fMRI (Campabadal et al., 2020).

### 3.9 Nodal SPL in iRBD

Nodal SPL was measured in the iRBD group using rs‐fMRI in two studies (Campabadal et al., 2020; Chen et al., 2022). In the patient group, Campabadal et al. (2020) observed no difference in this parameter between the two groups. In contrast, Chen et al. (2022) calculated nodal SPL in the patient group and realized that this parameter had increased in pre and postcentral gyrus, supramarginal gyrus, superior temporal gyrus, supra‐motor area, straight gyrus, middle cingulate gyrus and Rolandic operculum in iRBD patients, suggesting elevated segregation. The authors also found negative correlations between the SDMT score, TMT‐B, and nodal SPL.

### 3.10 Nodal DC in iRBD

The DC parameter was calculated in the iRBD group only in one study by Chen et al. (2022), who used rs‐fMRI and presented a decrease in this parameter in the limbic system, paracingulate gyrus, left right anterior cingulate gyrus, right cuneus, parietooccipital) left and right superior occipital gyrus and left and right middle occipital) after correction for multiple comparisons by Bonferroni's test. (Guo et al., 2018).

### 3.11 Hub distribution in iRBD

In the iRBD group, specific patterns of hubs were clearly reported in only one study by Park et al. (2019), who employed s‐MRI and showed that there were significant hub regions (mostly in the caudate nucleus and the frontal cortex) in iRBD patients compared to HC.

## 4 DISCUSSION

The aim study was to evaluate graph features at two local and global levels and to assess the potential of these metrics as brain biomarkers to identify the underlying physiopathology of iRBD group. With the rapidly growing use of graph features in neuroscience research, researchers use these features to analyze brain networks in iRBD patients. Given a range of 35%–50% incidence probability of RBD in PD and the recognition of this disorder as the PD initial phase, researchers are interested in identifying common brain biomarkers in these two diseases (Baumann‐Vogel et al., 2020). This section summarizes the main results from the reviewed studies and proposes recommendations for future investigations.

### 4.1 Summary and interpretation of graph disturbances in iRBD

#### 4.1.1 Measures of integration

Identical stability was not reported for integration in the reviewed studies. In the iRBD group, the results showed that there is a lower integration in one measurement (Wen et al., 2017). Moreover three findings (Chen et al., 2022; Park et al., 2019) reported higher integration to compare to HC. One of the major results found in this review was high changes in the findings, particularly in the results found for network integration. Therefore, there is a need for further research in this field using more sample size.

#### 4.1.2 Measures of segregation

A mix of the results revealed that four measurements found reductions in segregation in the iRBD group (Chen et al., 2022; Geng et al., 2022; Park et al., 2019). The occipital lobe and cerebellum in the iRBD group, respectively, compared to the HC group were the regions with the most changes in segregation. Additionally, three measurements indicated increases in segregation (Chen et al., 2022; Wen et al., 2017) in the iRBD group versus the HC group, and five measurements in some brain regions showed no differences (Campabadal et al., 2020; Lee et al., 2022; Wen et al., 2017). The following are the brain regions in which segregation increased in iRBD group.

- Occipital lobe especially the middle occipital gyrus and left lingual gyrus in the iRBD group compared to HC

Segregation might be the most important stable parameter in iRBD group in comparison with HC in this review. These measurements included CC, LE, and nodal SPL.

#### 4.1.3 Measures of centrality

The results of centrality measurements were not equally stable. Compared to HC, differences between patients in the iRBD group were shown in four measurements (reductions and elevations in every two measurements) (Campabadal et al., 2020; Chen et al., 2022; Lee et al., 2022; Park et al., 2019). In the following brain areas, both the decrease and increase of the centrality were observed:

- Increased centrality in the frontal lobe and caudate nucleus, olfactory cortex, temporal lobe, thalamus and cerebellum in the iRBD group compared to the HC group.
- Decreased centrality in the cingulate gyrus, parietooccipital region, and occipital lobe in the iRBD group compared to HC

According to these results (regarding brain regions), the difference between the two patient groups and HC depends on the brain area. Based on individual studies, the groups were different in structural BC, functional BC, structural DC, and functional DC.

#### 4.1.4 Hub disruption

This index indicates network flexibility upon damage and represents the global shift on the nodal scale. Hubs actually refer to high‐centrality nodes deeply affecting network topology estimating by several methods, including nodal degree centrality and nodal betweenness centrality calculation.

Compared to HC, hub disruption was observed in high‐BC areas in iRBD group (Park et al., 2019) including the following brain areas (see Table 2):

- Caudate nucleus and frontal cortex in the iRBD group

In this study, differences between the two groups were discovered for both reviewed centrality parameters (BC and DC) in structural connectivity and functional connectivity. The presence of high‐centrality regions in the patient groups versus the HC group indicates more decreased of integration in the former than in the latter. Further studies seem to be necessary for this field considering the importance of this distribution and the confusing results obtained for both groups.

#### 4.1.5 Correlation between graph parameters and neuropsychological data

The analysis of brain networks using graph theory is a robust tool to quantify brain topological features of iRBD patients using s‐MRI, DWI, and rs‐fMRI. In the reviewed studies, the correlation between neuropsychological data and graph parameters indicated that the highest correlation was found in nodal LE (directly) (Chen et al., 2022; Geng et al., 2022) and nodal SPL (Chen et al., 2022) (negatively) in the iRBD group. In other studies, no correlation was detected between these data and graph parameters. The importance of iRBD as the primary phase of Parkinsonism necessitates further investigations on the correlation between these data and graph parameters in the future.

### 4.2 Different changing patterns in iRBD

In the reviewed studies, high divergence was generally observed in the reported results. This may imply that brain graphs do not estimate the same results depending on the imaging technique and the brain connectivity matrix (structural connectivity and functional connectivity). Different imaging techniques and connectivity matrices provide contrasting information about brain structure and function. s‐MRI produces information about the morphology of brain gray matter whereas DWI provides information about structural connectivity between brain areas, and rs‐fMRI creates information during automated brain activity based on the BOLD signal measurement. Combined information obtained from different imaging techniques is generally problematic. For example, functional connectivity has been shown to be present in areas where structural connectivity is low or absent whatsoever (Wang et al., 2021), and there is a similar trend in the correlation between cortical thickness and WM fiber connection (Gong et al., 2012).

In addition, significant effectors in contrasting results are confounding factors, the most important of which may be the lack of a standard method for graph production. As indicated in previous study (Yeh et al., 2021), parcellation templates can influence the values of graph parameters and should be considered upon graph comparisons given that various parcellations (90–246 in size) had been used in the reviewed studies (Table 2). Another important factor of consideration is the diversity of cortex and subcortex areas in the parcellation of different templates, causing differences in the signal‐to‐noise ratio (Yeh et al., 2021). Brain gyri and sulci patterns have many changes in different subjects, which complicates the correct map of templates between different subjects in a study.

Using graph analysis, a network can be formed by binary and weighted methods (Tables 3 and 4). The former often considers a threshold for connectivity edges, and weak connectivity levels are not considered in the analysis because of shortening the calculation time, noise removing and obtaining reliable results (Yeh et al., 2021). Importantly, binary networks consider connectivity levels higher than a threshold; therefore, they do not show minor changes in the network, and over 90% removal of weak connectivity levels may lead to complications for the statistical analysis (Civier et al., 2019). In an imaging method, the type of edges between nodes may also be considered differently. In DWI, for example, connectivity may be determined based on mean diffusivity (MD) and fractional anisotropy (FA) parameters, the number of fibers, and the length of fibers between each brain area (Table 4). Consequently, this factor creates different structural connectivity matrices and may produce conflicting results.

The study type, or more correctly, the type of analyzed data, aging, and different pathological conditions including treatment of patients with drugs and comorbidity are the other factors affecting graph parameters. These factors may also influence the instability of results (Zhang et al., 2020). As reported in the introduction, iRBD may be the primary phase of α‐synucleinopathies, particularly PD. Thus, pathological conditions (disease severity and comorbidity) and age can be the major effectors (Zhang et al., 2020).

Due to large divergencies in integration, the results of reviewed studies were evaluated methodologically (e.g., imaging technique, toolbox, sample size, and the average age of samples) in iRBD group (Table 3). Differences were found in the imaging technique, image processing, sample size, and toolbox used in studies, which were compared individually in the following. In terms of DWI, integration parameters were evaluated methodologically in iRBD group. Lee et al. (2022) used the DSI studio for the graph analysis while Wen et al. employed the brain Connectivity toolbox. Besides, compared to latter study (Wen et al., 2017), the former (Lee et al., 2022) used more homogenous data in comparison to second (related to a single center versus PPMI data) and fully different toolbox types for data analysis (Table 3).

### 4.3 Methodological issues and future perspectives

The use of the graph theory for network disorder analysis is still in its infancy, which necessitates more studies to address unanswered questions in this field to demonstrate the extent to which this disease affects brain neural networks. Moreover, the network analysis enables mapping comprehensively brain connectivity, but models defined to interpret the graph network using integration, segregation, and centrality are in their infancy. It is also noteworthy that the network analysis provides information about voxels and areas, without considering the focal information of individual elements. Other information, such as brain gray matter morphology, cerebral cortex thickness, and spatial activation of brain areas, can also be extracted from s‐MRI, DWI, and rs‐fMRI images. A combination of these data and network information can provide researchers with more in‐depth knowledge of brain functions.

Furthermore, there are more challenges of consideration by researchers concerning the network analysis in iRBD. Since human brain connectivity includes a complex set of neurons and their dependent elements, it is highly essential to accurately define nodes and the connectivity between them to show a standard method for network analysis. Nodes can be defined using different templates, including anatomical templates, functional templates, etc. Therefore, information should be compared more cautiously by introducing the type of applied template. It is critical to select the template type according to the studied disease, and a parcellation should be selected that contains the areas involved in iRBD disease.

Moreover, the multitude number of neurons (billions) and their highly abundant connectivity urge the pressing need for progress in computer systems and imaging techniques. Along with selecting an appropriate template, it is also important to accurately define brain connectivity or edges in brain networks. In s‐MRI methods, connectivity between different areas is typically defined using morphological connectivity or covariance patterns, such as cerebral cortex thickness or volume. In DWI, on the other hand, the number of brain fibers, the length of fibers, FA, MD, AD, and RD may be used to define edges. In functional connectivity studies, edges or connectivity are determined using correlation coefficients in a time range or coherence measurements in the frequency range. The binary or weighted‐based selection of edges or connectivity can also affect the results. The morphological organizations of the network are also influenced by the thresholding type of edges.

The data type used for image acquisition, preprocessing, and processing is also another factor causing different and divergent results. More different results are generally produced in studies conducted using homogenous data in one or more limited centers than data in such databases as Parkinson's Progression Markers Initiative (PPMI). It is recommended to compare data in similar conditions with a specific imaging technique.

Another related factor worthy of note is the diagnostic method used for iRBD patients. To diagnose patients, the golden standard is to use polysomnography whereas RBDSQ is used to analyze the disease for inhomogeneous data, such as the PPMI database, and this factor can also produce high differences in results.

## 5 CONCLUSIONS

No standard method has so far been established to define graph networks in the iRBD disease. The divergent results in this study demonstrate that brain graphs are heavily dependent on the imaging technique type and the analysis method of imaging data. This highlights the need for more studies using multimodality imaging in this field.

The use of the graph theory makes it possible to characterize different brain areas and their roles in various networks. Further multimodality studies will disclose more details on brain connectivity in the patient group and normal individuals.

## AUTHOR CONTRIBUTIONS

**Milad Najafzadeh**: Conceptualization; investigation; methodology; visualization; software; formal analysis; writing—original draft. **Fatemeh Mohammadian**: Investigation; methodology. **Sara Mirabian**: Investigation; methodology. **Zohre Ganji**: Methodology. **Hossein Akbari**: Methodology; Writing—original draft. **Masoud Rezaie**: Methodology; writing—original draft. **Esmaeil Ranjbar**: Methodology. **Hoda Zare**: Conceptualization; investigation; methodology; writing—original draft; writing—review and editing; supervision; visualization. **Shahrokh Naseri**: Investigation; writing—original draft; validation; methodology; writing—review and editing; supervision. **Luigi Ferini‐Strambi**: Conceptualization; investigation; writing—review and editing; methodology.

## CONFLICT OF INTEREST STATEMENT

The authors declare that they have no conflict of interest.

### PEER REVIEW

The peer review history for this article is available at https://publons.com/publon/10.1002/brb3.3460.

## Data Availability

The data that support the findings of this study are available from the corresponding author upon reasonable request.
